# Changes of hemodynamic and cerebral oxygenation after exercise in normobaric and hypobaric hypoxia: associations with acute mountain sickness

**DOI:** 10.1186/s40557-018-0276-2

**Published:** 2018-11-19

**Authors:** Tobias Kammerer, Valentina Faihs, Nikolai Hulde, Andreas Bayer, Max Hübner, Florian Brettner, Walter Karlen, Julia Maria Kröpfl, Markus Rehm, Christina Spengler, Simon Thomas Schäfer

**Affiliations:** 1 Department of Anaesthesiology, University Hospital, LMU Munich, Marchioninistr. 15, 81377 Munich, Germany; 2 Walter Brendel Centre of Experimental Medicine, LMU Munich, Marchioninistr. 15, 81377 Munich, Germany; 3 Exercise Physiology Lab, Institute of Human Movement Sciences and Sport, ETH Zurich, Winterthurerstr. 190, 8057 Zurich, Switzerland; 4 Mobile Health Systems Lab, Institute of Robotics and Intelligent Systems, ETH Zurich, Lengghalde 5, 8092 Zurich, Switzerland; 5 Institute of Anesthesiology, Heart and Diabetes Center NRW, Ruhr University Bochum, Georgstr. 11, 32545 Bad Oeynhausen, Germany

**Keywords:** Acute mountain sickness, Cerebral oxygenation, Near-infrared spectroscopy, Normobaric hypoxia, Hypobaric hypoxia, Cognitive dysfunction

## Abstract

**Objective:**

Normobaric (NH) and hypobaric hypoxia (HH) are associated with acute mountain sickness (AMS) and cognitive dysfunction. Only few variables, like heart-rate-variability, are correlated with AMS. However, prediction of AMS remains difficult. We therefore designed an expedition-study with healthy volunteers in NH/HH to investigate additional non-invasive hemodynamic variables associated with AMS.

**Methods:**

Eleven healthy subjects were examined in NH (FiO_2_ 13.1%; equivalent of 3.883 m a.s.l; duration 4 h) and HH (3.883 m a.s.l.; duration 24 h) before and after an exercise of 120 min. Changes in parameters of electrical cardiometry (cardiac index (CI), left-ventricular ejection time (LVET), stroke volume (SV), index of contractility (ICON)), near-infrared spectroscopy (cerebral oxygenation, rScO_2_), Lake-Louise-Score (LLS) and cognitive function tests were assessed. One-Way-ANOVA, Wilcoxon matched-pairs test, Spearman’s-correlation-analysis and Student’s t-test were performed.

**Results:**

HH increased heart rate (HR), mean arterial pressure (MAP) and CI and decreased LVET, SV and ICON, whereas NH increased HR and decreased LVET. In both NH and HH cerebral oxygenation decreased and LLS increased significantly. After 24 h in HH, 6 of 11 subjects (54.6%) developed AMS. LLS remained increased until 24 h in HH, whereas cognitive function remained unaltered. In HH, HR and LLS were inversely correlated (*r* = − 0.692; *p* < 0.05). More importantly, the rScO2-decrease after exercise in NH significantly correlated with LLS after 24 h in HH (*r* = − 0.971; *p* < 0.01) and rScO2 correlated significantly with HR (*r* = 0.802; *p* < 0.01), CI (*r* = 0.682; *p* < 0.05) and SV (*r* = 0.709; *p* < 0.05) after exercise in HH.

**Conclusions:**

Both acute NH and HH altered hemodynamic and cerebral oxygenation and induced AMS. Subjects, who adapted their CI had higher rScO2 and lower LLS. Furthermore, rScO2 after exercise under normobaric conditions was associated with AMS at high altitudes.

## Introduction

Acute hypoxia under both normobaric (NH) and hypobaric (HH) conditions is associated with symptoms of acute mountain sickness (AMS) and cognitive dysfunction in humans [1–4]. The degree of hypoxemia plays a central role in the pathophysiology of AMS [5]. However, decrease of peripheral oxygen saturation (SpO2) under hypoxic conditions has previously been shown to be of poor predictive value. Therefore, most publications identified a combination of different variables to predict AMS [6, 7]. Unfortunately, some of these variables are difficult to raise under laboratory conditions or must be measured invasively. Recently, heart rate variability (HRV) was identified as a potential predictor for AMS in healthy subjects, where the underlying mechanism is unclear [8]. Predicting the likelihood to develop AMS before ascent to HH could be important not only for mountaineers but also for untrained individuals as improved transport technologies allow to rapidly ascending to high altitude. This exposes also persons with potentially preexisting conditions like cardiovascular disorders to an increased risk for AMS. It is therefore of particular interest to find further non-invasive variables for AMS prediction. Simultaneously, exposure to high altitudes is associated with a decrease of cerebral oxygen saturation, which is controversially discussed in terms of the incidence of cognitive dysfunction [3, 9–11].

We therefore performed a study with healthy volunteers to identify non-invasive variables under NH as predictors for AMS. Using electrical cardiometry, near-infrared spectroscopy, cognitive function testing and Lake-Louis-Score (LLS) we hypothesized that 1.) NH and HH would lead to similar changes of hemodynamic variables, decreases in systemic (SpO2) and cerebral oxygen saturation (rScO2) and that 2.) hemodynamic changes and rScO2 in NH would correlate with the degree of AMS in HH.

## Materials and methods

### Subjects and experimental protocol

**Fig. 1:**
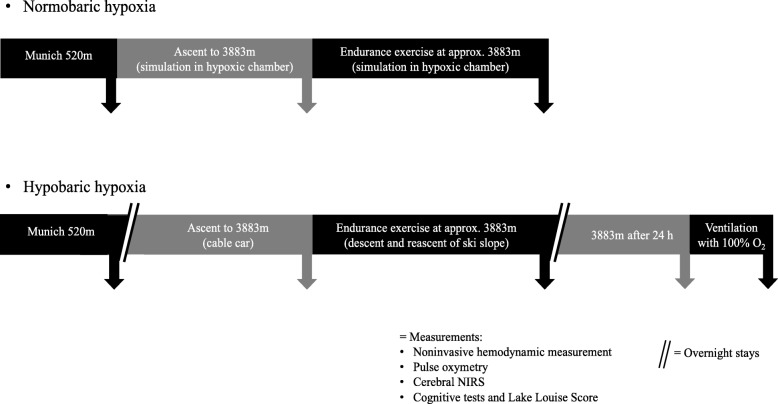
Experimental protocol for ascent and exercise in normobaric (hypoxic chamber) and hypobaric (Little Matterhorn summit) hypoxia

After approval by the local Ethics Committee of the University of Munich, Germany (project no. 350–16) and obtaining written informed consent, 11 healthy female (*n* = 5) and male (*n* = 6) individuals aged 36.4 (±7) years, with mean height of 178 (±6) cm and mean body mass index of 22.7 (±2) kg/m^2^, were included in the study. All subjects were in good physical and mental condition, without any comorbidities or medication and were measured at different time points in normobaric normoxia, NH and HH (see Fig. 1). All individuals did not stay at a height of more than 2000 m a.s.l. until at least 6 weeks before the study.

The following protocol was done to evaluate effects of hypobaric hypoxia: after initial baseline measurements in Munich at 520 m a.s.l. (normoxia), all individuals were transferred to Zermatt, Switzerland (1608 m a.s.l.) by car. Next morning, ascent to Little Matterhorn at 3883 m a.s.l. was done by cable car (duration 45 min), followed by further measurements. After this, all subjects performed 120 min endurance exercise by descending to around 3500 m and reascending to 3883 m a.s.l.. Immediately after physical exercise, measurements were performed in an expedition tent (Keron 4 GT, Hilleberg AB, Frösön, Sweden) on the glacier. After spending one night at 3883 m a.s.l. in the hut, measurements were performed again 24 h after arrival at high altitude and repeated after breathing 100% oxygen for 5 min (Fig. 1).

Additionally, 7 of these individuals (3 female, 4 male; 36.3 (±4) years; 179 (±6) cm; BMI 22.7 (±2) kg/m^2^) were examined under normobaric conditions in a hypoxic chamber (VPSA 16; Van Amerongen CA Technology, Tiel, Netherlands) 6 weeks before (*n* = 4) and 6 weeks after (*n* = 3) high altitude exposure. Again, baseline measurements were performed in Munich at 520 m a.s.l. (normoxia), followed by passive ascent (duration 45 min) to simulated 3883 m a.s.l. in the hypoxic chamber and 120 min of endurance exercise at simulated 3883 m a.s.l. including alternately cycling and walking with 15% slope (Trac 3000 Tour Med and Crosstrainer 3000; Ergo-Fit Inc., Pirmasens, Germany) (see Fig. 1). To simulate an altitude of 3883 m a.s.l., participants were exposed to an inspiratory oxygen fraction of 13.1% at constant room temperature (20–24 °C) and humidity (20–27%) for 4 h.

### Acute mountain sickness score and cognitive performance

Symptoms of AMS, consisting of headache, gastrointestinal problems, insomnia, fatigue and dizziness, were evaluated using a self-report questionnaire according to the Lake Louise Score (LLS, 5 items, maximum point sum 15) [12]. AMS after exposition to NH/HH was defined as presence of moderate or severe headache in combination with a LLS point sum of ≥3. Cognitive function was evaluated on an Android tablet with a test battery developed by the Mobile Health Systems Lab, Eidgenössische Technische Hochschule (ETH), Zurich, Switzerland, with a total of 4 different cognitive tests: first, Trail Making Test A (TMT-A), where subjects must connect numbers and Trail Making Test B (TMT-B), where subjects must connect numbers and letters in an ascending sequence (i.e. 1-A, 2-B, 3-C…) as quickly as possible. Second, a target reaction test (tRT) and a sorting reaction test (sRT) were performed. In the tRT, one must keep a finger on a predefined area of the tablet until a spot appears which should be touched as quickly and accurately as possible. In the sRT, similar looking geometrical forms must be quickly touched in the order displayed above. For all cognitive tests, speed, accuracy and response time were recorded electronically. Subjects were asked to take 3 of each test type in an isolated environment. Prior to the study, individuals trained all tests to become familiar with the test battery and handling of the tablet.

### Cerebral oxygenation and advanced hemodynamic monitoring

All variables regarding hemodynamics, peripheral oxygen saturation and cerebral oxygenation were repeated five-times at each time point to calculate mean values for every subject. Heart rate, peripheral oxygen saturation and non-invasive blood pressure were measured with a mobile battery powered monitoring system (Infinity® M540 Monitoring, Draeger Inc., Luebeck, Germany). Cerebral oxygenation (rScO_2_) was measured using a noninvasive near-infrared spectroscopy (NIRS) monitor (INVOS™ 5100C Cerebral/Somatic Oximeter, Covidien G, Boulder, CO, USA) powered by battery and a portable 240-V power converter. Non-invasive advanced hemodynamic monitoring was performed with a portable monitor using electrical cardiometry (ICON™ Cardiac Output Monitor, Osypka Medical GmbH, Berlin, Germany) to measure cardiac index (CI), stroke volume (SV), index of contractility (ICON) and left-ventricular ejection time (LVET). This technique is based on variations of thoracic electrical bioimpendance due to changes in thoracic conductivity during the heart cycle registered by highly conductive sensors (Cardiotronic Sensors™ Osypka Medical GmbH, Berlin, Germany) [13].

### Statistical analysis

Normally distributed data are given as mean and standard deviation. In case of repeated measurements, a one-way ANOVA with Greenhouse-Geisser correction, followed by multiple comparisons with Bonferroni correction was performed (*p* < 0.05/n). Differences of LLS were analyzed by Wilcoxon matched-pairs test. Correlations were assessed using Spearman’s rank correlation coefficients. T-test with Bonferroni-Sidak correction was used to detect differences between groups after exercise. All statistical analyzes were performed using PRISM version 7 (GraphPad Software Inc., La Jolla, CA, USA).

## Results

**Fig. 2:**
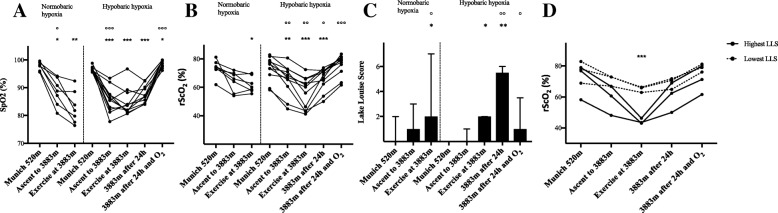
Peripheral and cerebral oxygen saturation and Lake Louise Score in normobaric (hypoxic chamber; *n* = 7) and hypobaric (Little Matterhorn summit; *n* = 11) hypoxia. **a** Changes in pulse oximetry saturation (SpO_2_: %). **b** Changes in regional cerebral oxygen saturation (rScO_2_ % mean values of right and left hemisphere). Statistical analysis for A and B using one-way repeated-measures ANOVA with Greenhouse-Geisser correction, followed by multiple comparisons with Bonferroni correction. **c** Changes in Lake Louise Score (LLS: median and interquartile-range) for evaluation of Acute Mountain Sickness. Subjects completed a self-reported questionnaire on a tablet. Statistical analysis using Wilcoxon matched-pairs signed rank tests. **d** Comparison of regional cerebral oxygen saturation (rScO_2_: %; mean values of right and left hemisphere) between the four subjects with lowest LLS and the four subjects with highest LLS after 24 h in hypobaric hypoxia. Statistical analysis using t-test with Bonferroni-Sidak correction, difference between groups after exercise * *p* < 0.05, ** *p* < 0.01, *** *p* < 0.001 vs. baseline in Munich at 520 m; ° *p* < 0.05, °° *p* < 0.01, °°° *p* < 0.001 vs. prior time point

**Fig. 3:**
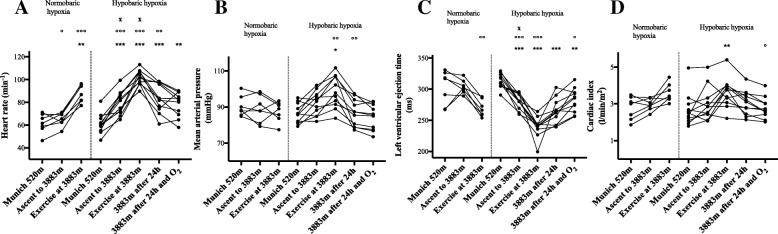
Hemodynamic variables in normobaric (hypoxic chamber; *n* = 7) and hypobaric (Little Matterhorn summit; *n* = 11) hypoxia. Statistical analysis using one-way repeated-measures ANOVA with Greenhouse-Geisser correction, followed by multiple comparisons with Bonferroni correction. **a** Changes in heart rate (HR; min^− 1^). **b** Changes in mean arterial pressure (MAP; mmHg). **c** Changes in left ventricular ejection time (LVET; ms). **d** Changes in cardiac index (CI; l/min/m^2^). * *p* < 0.05, ** *p* < 0.01, *** *p* < 0.001 vs. baseline in Munich at 520 m; ° *p* < 0.05, °° *p* < 0.01, °°° *p* < 0.001 vs. prior time point; **x** *p* < 0.05 between corresponding time points in hypobaric and normobaric hypoxia (*n* = 7)

Compared to baseline, systemic and cerebral oxygen saturation after exercise decreased significantly under normobaric and hypobaric conditions (NH: SpO_2_ 97.7 ± 1.5% versus 82.9 ± 5.8%, *p* < 0.01; rScO_2_ 73.7 ± 6.0% versus 62.0 ± 5.6%, p < 0.05; HH: SpO_2_ 96.7 ± 1.0% versus 84.8 ± 4.9%, *p* < 0.001; rScO_2_ 73.4 ± 8.3% versus 56.3 ± 11.0%, *p* < 0.001) (see Fig. 2a and b). After 24 h in hypobaric hypoxia 6 of 11 subjects (54.6%) developed AMS (moderate or severe headache in combination with LLS point sum ≥3). LLS point sum increased significantly after exercise in NH and HH and highly significantly after 24 h in HH compared to baseline measurements (see Fig. 2c). However, cognitive function tests at either NH or HH remained unchanged and did not correlate with changes in hemodynamics, oxygen saturation or LLS. Comparing the four subjects with highest and lowest LLS in HH, significant differences in rScO_2_ between groups were revealed (*p* < 0.01; see Fig. 2d). During exercise HR was 133.3 ± 16.9 min^− 1^ in NH compared to 142.8 ± 20.5 min^− 1^ in HH (*p* = 0.002). After exercise, HR remained increased in both NH (61.6 ± 8.5 min^− 1^ versus 87.7 ± 7.8 min^− 1^; *p* < 0.01) and HH (61.1 ± 8.8 min^− 1^ versus 101.8 ± 6.8 min^− 1^; *p* < 0.001) compared to baseline measurements, accompanied by a simultaneous decrease of LVET whereas mean arterial pressure only increased in hypobaric conditions (86.6 ± 5.4 mmHg versus 98.8 ± 8.9 mmHg; *p* < 0.05) compared to baseline (see Figs. 3a, b and c). Cardiac index increased significantly after exercise at high altitude (see Fig. 3d). Further values are given at Table 1. In HH, a significant negative correlation was found between HR and LLS (*r* = − 0.692; *p* < 0.05). Additionally, after exercise, rScO2 correlated with HR (*r* = 0.802; *p* < 0.01), CI (*r* = 0.682; *p* < 0.05) and SV (0.709; *p* < 0.05). Furthermore, in HH, rScO_2_ after exercise showed an inverse correlation with LLS after 24 h (*r* = − 0.817; *p* < 0.01). Most importantly, reduced rScO2 after exercise in NH was inversely correlated with LLS after 24 h on the mountain (*r* = − 0.971; *p* < 0.01).

**Table 1:** Parameters measured with noninvasive hemodynamic monitoring using Electrical Cardiometry

	Normobaric hypoxia (hypoxic chamber)			Hypobaric hypoxia (Little Matterhorn summit)				
	Munich 520 m	Ascent to simulated 3883 m	Exercise at simulated 3883 m	Munich 520 m	Ascent to 3883 m	Exercise at 3883 m	3883 m after 24 h	3883 m after 24 h and O_2_
Cardiac index (l/min/m^2^)	2.8 ± 0.6	3.0 ± 0.3	3.6 ± 0.50	2.7 ± 0.9	3.0 ± 0.9	3.6 ± 0.8**	3.1 ± 0.7	2.8 ± 0.6
Cardiac output (l/min)	5.2 ± 0.8	5.6 ± 0.5	6.8 ± 0.8	5.0 ± 1.5	5.8 ± 1.6	6.9 ± 1.5	5.8 ± 1.4	5.3 ± 1.2
Stroke volume (ml)	84.3 ± 7.8	85.3 ± 7.6	76.5 ± 8.4	81.4 ± 14.0	74.1 ± 14.0	68.0 ± 10.7*	67.6 ± 13.5*	69.6 ± 13.6
Left ventricular ejection time (ms)	303 ± 27	304 ± 11	267 ± 13	313 ± 12	281 ± 12***	240 ± 16***	264 ± 20***	282 ± 19**
Index of contractility	68.8 ± 20.1	68.2 ± 8.0	53.1 ± 13.8	64.8 ± 22.6	50.0 ± 17.5	45.2 ± 17.1**	44.0 ± 15.2**	45.9 ± 13.6*

Values are presented as mean ± SD. Statistical analysis with one-way repeated-measures ANOVA with Greenhouse-Geisser correction, followed by multiple comparisons with Bonferroni correction for multiple comparisons, *p < 0.05, **p < 0.01, ***p < 0.001 vs. baseline in Munich at 520 m

## Discussion

Acute mountain sickness is an ongoing topic in high altitude medicine. Until now, different variables with a predictive value for AMS could be identified [6, 7]. Of high interest, Sutherland et al. have recently shown a significant correlation between heart rate variability and AMS, evaluated by LLS [8]. However, HRV can be of limited value in subjects with cardiovascular comorbidities i.e. arrhythmias or ß-blocker intake. It is therefore of interest to identify further predictive variables which can be assessed easily even in remote areas. In our study we were able to identify further hemodynamic variables associated with AMS. To the best of our knowledge, this is the first trial using electrical cardiometry combined with cerebral near-infrared spectroscopy in high altitude.

In detail, in our healthy volunteers we have seen significant increases in HR, CI, SV and decreases in LVET, ICON and cerebral oxygenation. Effects on hemodynamic variables were most pronounced after exercise in both, normobaric and hypobaric hypoxia at an altitude of 3883 m a.s.l.. In accordance to the results of Sutherland et al. [8] we found a significant negative correlation between HR and LLS in hypobaric conditions. However, variables assessed in hypobaric conditions could only allow recommendations to interrupt further ascent or to immediately descend but they cannot provide predictive value. In contrast, associations between variables assessed in a safe normobaric training environment and the risk of developing actual AMS at high altitude could help to predict the individual’s risk for AMS. In this regard, we could show a negative correlation between normobaric rScO2 decrease after exercise and LLS after 24 h in hypobaric conditions on the mountain. Due to the fact that our subjects were the same under NH and HH, an association is likely. Thus, the rScO2 decrease in simulated altitude in a normobaric chamber might serve as a predictive variable in the future. This is interestingly due to the fact, that there is actually an existing debate about the air equivalent model, which points out, that NH and HH are two different stimuli for AMS [14, 15]. In some previously published trials, the severity of AMS was higher in HH than in NH whereas the underlying mechanism is unclear [16, 17]. Additionally, preacclimatization in HH can reduce severity of AMS whereas preacclimatization in NH was less effective [18–21]. However, the main factor affecting AMS seems to be acclimatization to hypoxia. The role of hypoxia in AMS was also supported by our presented data. When focusing on the four individuals with highest and lowest LLS, it turns out that they had lowest / highest cerebral oxygenation which supports the hypothesis, that oxygen delivery and clinical symptoms are associated. Simultaneously, the significant correlation between the rScO2 decrease after exercise and corresponding cardiac index underlines the importance of adequate hemodynamic adaptation to hypoxic conditions. Although this is not a new finding, it is of interest that we have found associations between short exercise in a hypoxic chamber and symptoms of AMS in high altitude. Furthermore, subjects who were able to adequately adapt their cardiac index either by an increase of HR or SV, have shown better cerebral oxygenation and lower LLS point sum. Thus, rScO2 after exercise under normobaric hypoxia could be a possible predictor for AMS at high altitude. This could be interestingly due to the fact that access to high altitude areas is getting easier even for subjects with cardiovascular diseases exposing those individuals at risk for AMS [22].

In contrast to the observed changes in hemodynamics and oxygenation, the cognitive function tests used in this trial did not reveal any changes. This is in some accordance with the literature where results are inconsistent: Asmaro et al. (2013) investigated cognitive dysfunction in hypoxic conditions at a simulated altitude up to 7.620 m a.s.l. in healthy volunteers [2]. The authors were able to detect impairments of cognitive performance in this setting of extreme high altitude. Davranche et al. (2016) studied brain oxygenation and cognitive function during 4 days at an altitude of 4.350 m a.s.l. and detected a reduction in terms of speed and accuracy in the early phase of hypoxic exposure whereas the slowdown of reaction time was not detectable anymore after 2 days at high altitude [3]. However, Issa et al. (2016) found no significant changes in overall cognitive performance during an expedition to Mount Everest [23]. Also, Pramsohler et al. (2017) described inconsistent finding regarding cognitive function in subjects that slept at a simulated altitude of 5.500 m a.s.l. [1]. While the combined parameter of cognitive- and motoric reaction time didn’t change, these authors even found a correlation between lower SpO_2_ and shorter cognitive reaction time. In summary, the data regarding hypoxia and cognitive function are contradictory. One reason for this could be the fact that the tests applied throughout the studies are not standardized and vary. In any case, at this point, cognitive function tests are not associated with symptoms of AMS.

Our study has limitations: First, we only included healthy volunteers and can only speculate that the cerebral oxygenation decrease in normobaric hypoxia would be of predictive value in patients with decreased heart rate variability. Secondly, due to the higher heart rate, exercise intensity seems to be slightly higher in HH than in NH. This is probably due to the fact that the expedition on the glacier had not been carried out as originally planned due to the weather conditions, but had to be modified. Third, the set of cognitive function tests used was insensitive to detect mild cognitive impairment. Thus, in future studies, a larger set of more standardized tests is recommended. However, our trial provides new insights regarding the relation between hemodynamics, cerebral oxygenation and LLS, and thus these variables assessed in normobaric conditions might help to predict AMS in high altitude.

## Conclusion

Non-invasive hemodynamic variables and cerebral oxygenation after exercise in normobaric hypoxia seem to be associated with the occurrence of acute mountain sickness at high altitude. This could be particularly interesting as a predictor for acute mountain sickness. The variables described here for the first time should therefore be investigated further in high altitude including more healthy participants as well as subjects with comorbidities.
